# Hybrid Microtubule–Solid‐State Nanopores for Single‐Molecule Analysis

**DOI:** 10.1002/elps.70091

**Published:** 2026-03-29

**Authors:** Matthew O'Donohue, Chaoming Gu, Byungsoo Kim, KeunMin Ken Lee, Sangyoup Lee, Chi Won Ahn, Myung Chul Choi, Min Jun Kim

**Affiliations:** ^1^ Department of Mechanical Engineering Southern Methodist University Dallas Texas USA; ^2^ Depatment of Bio and Brain Engineering Korea Advanced Institute of Science and Technology Daejeon Republic of Korea; ^3^ Bionic Research Center, Biomedical Research Division Korea Institute of Science and Technology Seoul Republic of Korea; ^4^ Nanomaterials Technology Development Center National Nanofab Center at KAIST Daejeon Republic of Korea

**Keywords:** bioelectronic nanodevice, ion transport, microtubule, microtubule–solid‐state nanopore, single‐molecule analysis

## Abstract

We demonstrate a hybrid microtubule–solid‐state nanopore (MT–SSN) platform that enables label‐free single‐molecule analysis. Under continuous voltage bias, individual taxol‐stabilized MTs are electrostatically anchored into an SSN to form a stable conduit for ionic current. We measured ionic current through MT–SSNs under two distinct configurations, one in which the SSN constricts ionic flow, and the other where the MT itself serves as the primary conduction channel. The geometrical asymmetry of the hybrid MT–SSN leads to a pronounced current–voltage asymmetry, that is, current rectification. Compared to bare SSNs, the hybrid MT–SSN slows double‐stranded DNA translocation by up to ∼3.5× and enhances event‐level signal contrast by increasing the relative current blockade, despite an increase in baseline low‐frequency noise. By repurposing the hollow, charged nanotubule of MTs to establish a novel framework for probing nanoscale ionic transport at the single‐molecule level, this study provides insight into the broader use of cytoskeletal proteins for bioelectronics sensing.

## Graphical abstract

Nanopores electrically detect molecules during translocation, but short passage times limit resolution. By anchoring a microtubule to a solid‐state nanopore, this hybrid system significantly prolongs DNA translocation while enabling simultaneous measurement of microtubule electrical properties, advancing nanoscale sensing and bioelectronic interrogation.

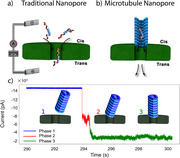

**Abbreviations**
CT‐CDB: chemically‐tuned controlled dielectric breakdown
dsDNA: double‐stranded DNA
*I*–*V*
: current–voltage curve
MT: microtubule
MT–SSN: microtubule–solid‐state nanopore
SSN: solid‐state nanopore

## 1 Introduction

Nanopore resistive–pulse sensing detects modulation of ionic current through a narrow opening that separates two electrolyte‐filled chambers. Solid‐state nanopores (SSNs), introduced in the early 2000s as alternatives to biological nanopores used in next‐generation DNA sequencing [1, 2, 3, 4, 5, 6, 7, 8], offer advantages, such as tunable pore diameters and surface functionalization. However, SSNs are often limited by higher excess noise and rapid translocation times [9], which can obscure transient features and hinder discrimination of structurally similar or weakly interacting molecules. Strategies that increase dwell time and event‐level signal contrast are therefore critical for resolving fast dynamics and low‐amplitude signals in single‐molecule electrophoretic measurements. To address these limitations, researchers have developed hybrid systems combining solid‐state and biological nanopores [10, 11, 12]. For example, Keyser et al. and Joty et al. demonstrated the electrophoretic anchoring of DNA origami onto an SSN using a continuous voltage bias [10, 12], and a hybrid α‐hemolysin/SSN has been successfully used for single‐molecule studies [11].

Microtubules (MTs) are hollow cylindrical protein nanotubes with average inner and outer diameters of approximately 15 and 25 nm, respectively [13, 14]. MTs are composed of globular dimeric αβ‐tubulin subunits, which polymerize head‐to‐tail into linear chains called protofilaments. The protofilaments laterally associate to form a lattice structure. Approximately 13 protofilaments are arranged in a helical lattice to form the cylinder of the MT [15]. MTs are rigid, with persistence lengths typically ranging from 1 to 10 mm [16, 17, 18, 19]. MTs are anionic with a net charge of tubulin of 41e. Due to their robust structure, MTs have been employed in various nanodevices [20], including piconewton force meters [21] and nanoscale bridges suspended across pillars [22]. Therefore, MTs offer a unique opportunity as rigid, hollow, and highly charged biological nanotubes capable of imposing strong steric and entropic constraints without chemical modification.

In this study, we repurposed a native MT for a completely novel sensing function by implementing MTs into hybrid biological‐SSNs (MT–SSNs). By anchoring taxol‐stabilized MTs into SSNs, we measured ionic transport through the MT lumen and translocated double‐stranded DNA (dsDNA) across the hybrid system with significantly improved signal‐to‐noise ratios (SNRs).

**FIGURE 1:**
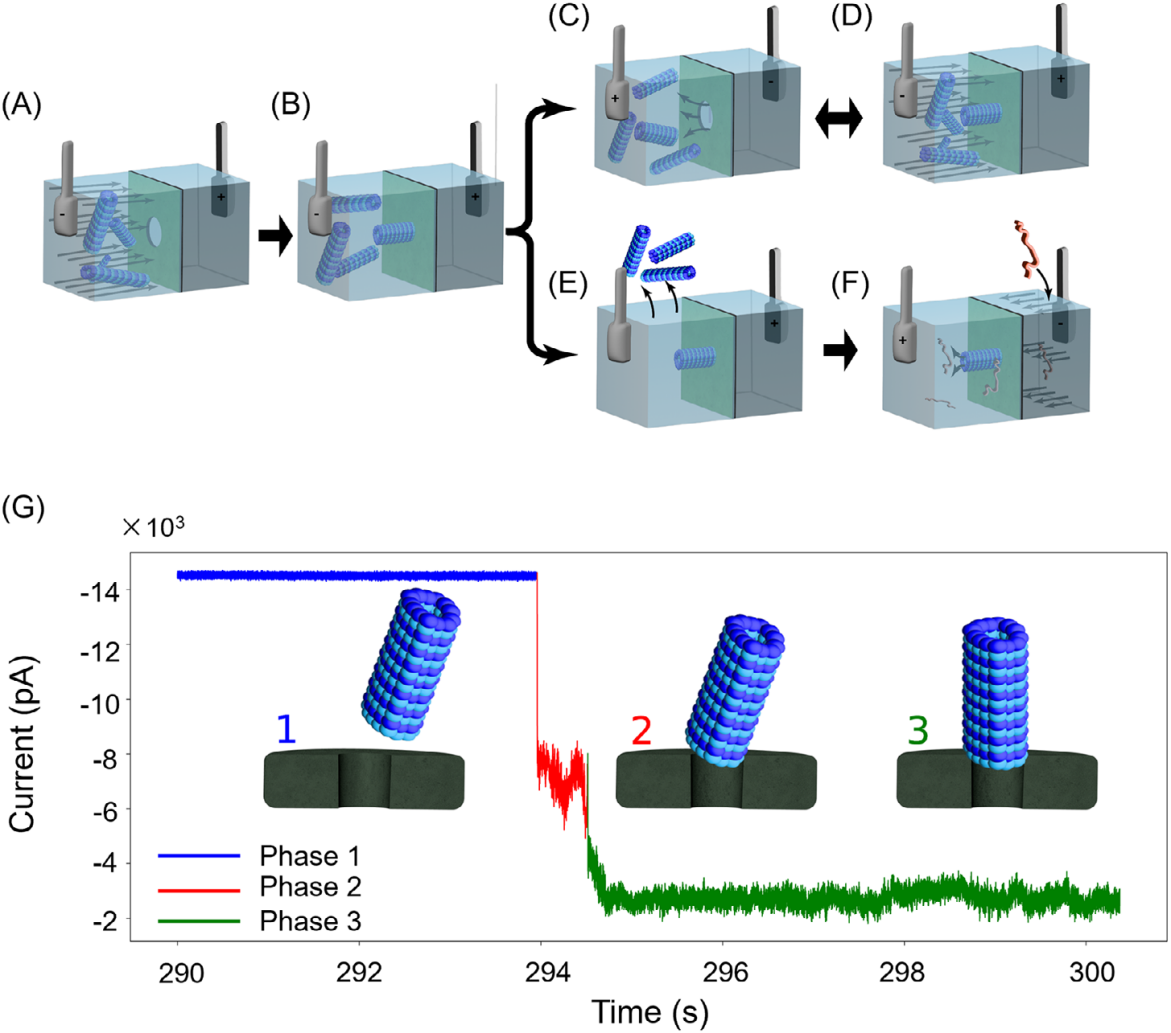
Schematics illustrating the experimental setup and processes for forming and utilizing the hybrid MT–SSN. (A and B) MTs are introduced into the chamber, and a voltage is applied to drive the MTs to the SSN, resulting in an MT anchored to the SSN. (C–F) Two pathways are shown: once an MT is inserted, a rapid voltage reversal detaches the MT from the SSN (C). Subsequent MT anchoring events can be initiated (D). Alternatively, the MT is allowed to stabilize and the free MTs are removed (E), and analyte is passed through the hybrid MT–SSN (F). (G) Current trace showing the characteristic steps of MT insertion in SSN. Phase 1 (blue) is the baseline current of the open‐pore prior to MT insertion. Phase 2 (red) is the intermediate/unstable MT anchoring phase. Phase 3 (green) is the stable MT anchoring to the SSN. Insets depict corresponding structural configurations of the microtubule in each phase.

Figure 1 shows the schematics illustrating the MT–SSN system. MTs are electrophoretically driven toward the SSN (Figure 1A). The radially symmetric structure of MTs results in an intrinsic electrostatic polarity along the MTs axis [23], allowing MTs to align directionally when subjected to an electric field [24, 25, 26, 27]. Driven by the steep electrical potential gradient concentrated at the SSN and its intrinsic charge polarity, the MT is guided to the SSN via electrostatic forces. Once at the SSN, the MT aligns above the pore and becomes constricted by the pore surface (Figure 1B). Under a continuous voltage bias, it remains electrokinetically anchored at the SSN entrance.

## 2 Materials and Methods

### 2.1 MT Assembly

Taxol‐stabilized MTs [28] were assembled using purified porcine brain tubulin (Cytoskeleton Inc.) under optimized conditions to ensure consistent polymerization. Tubulin stock solutions were prepared by reconstituting 1 mg of tubulin protein in 100 µL of ice‐cold PEM buffer (80 mM PIPES, 0.5 mM EGTA, 2 mM MgCl_2_, pH 6.9) to a final concentration of 10 mg/mL. The solution was aliquoted into 10 µL portions, snap‐frozen in liquid nitrogen, and stored at −70°C until further use.

Before polymerization, aliquots were thawed in a room temperature water bath for 1 min, immediately placed on ice, and diluted to 1 mg/mL by adding 90 µL of G‐PEM buffer (PEM supplemented with 1 mM GTP). G‐PEM was prepared fresh before each experiment by adding 10 µL of 100 mM GTP stock solution to 1 mL of ice‐cold PEM. Because GTP hydrolyzes rapidly, the buffer was kept on ice and used within 2 h.

To remove aggregates, the 1 mg/mL tubulin solution was centrifuged at 14 000 × *g* for 10 min at 4°C. The supernatant was carefully transferred to a new microtube on ice, and the final volume was recorded. A 2 mM taxol stock solution was prepared by dissolving taxol in 100% DMSO. The working solution was made by diluting 11 µL of 2 mM taxol stock into 100 µL of a 10:90 (v/v) DMSO:PEM mixture, yielding a final concentration of approximately 200 µM.

Tubulin polymerization was initiated by incubating the centrifuged tubulin solution at 37°C, followed by the stepwise taxol addition. First, 1 µL of the working taxol solution was added to the tubulin solution and was incubated for 5 min. After 5 min, an additional 10 µL of the taxol working solution was added, gently vortexed, and incubated for an additional 15 min at 37°C. The total polymerization time was 20 min.

### 2.2 Nanopore Fabrication

Nanopores were fabricated using the chemically tuned‐controlled dielectric breakdown (CT‐CDB) method, as described previously [29, 30, 31]. Free‐standing silicon nitride membranes with a thickness of 12 ± 2 nm (Norcada Inc.) were used as the substrate for all nanopore fabrication. For Regime 1 experiments, in which the MT diameter exceeded the nanopore diameter, nanopores were fabricated with diameters of 10 ± 1 nm. For Regime 2 experiments, where the MT diameter was smaller than the nanopore diameter, nanopores with diameters of 16 ± 1 nm were used.

Following fabrication, current–voltage (*I*–*V*) curves were recorded to confirm ohmic behavior and validate the pore's electrical characteristics. Baseline ionic currents were also measured across the full range of experimental voltages to verify the absence of contaminants or blockages that could lead to false‐positive events. All experiments were conducted using a 1 M KCl solution buffered with 10 mM Tris (pH 7.6).

### 2.3 Data Acquisition

Ionic current signals during nanopore experiments were recorded using an Axopatch 200B amplifier (Molecular Devices). Signals were digitized with a Digidata 1550B digitizer and filtered using a low‐pass Bessel filter with a 10 kHz cutoff frequency. Data acquisition was performed at a sampling rate of 250 kHz. Initial event detection and pulse characterization were carried out using EventPro 3.0 software [32]. Further data processing and analysis were performed using a custom Python script developed in‐house.

## 3 Results and Discussion

### 3.1 Hybrid MT–SSN System

Hybrid MT–SSN devices were reproducibly assembled across multiple experimental sessions, with more than 20 successful MT anchoring events supporting stable ionic current measurements (see Section S3 for representative examples). A nanomolar concentration of MTs was introduced into one chamber of a custom‐designed flow cell (Figure 1A) and electrophoretically driven toward a single SSN. The applied electric field facilitated the alignment and anchoring of MTs above the SSN (Figure 1B). The anchoring of MT to the SSN is marked by a stepwise decrease in ionic current (Figure 1G).

Once anchored, the MT could be ejected by rapidly reversing the applied voltage (Figure 1C). Subsequent anchoring events could then be initiated by reapplying the voltage (Figure 1D). However, if the voltage reversal was not applied promptly (within approximately 5 s), the MTs became stabilized and could no longer be ejected by reversing the voltage. Once an MT was stabilized, the free MTs could be removed from the solution (Figure 1E), and analyte could be translocated through the hybrid MT–SSN (Figure 1F).

Figure 1G depicts a current trace showing the characteristic steps of MT anchoring to the SSN. Phase 1 (blue) is the open‐pore baseline current before any MT engages the SSN. Some MT anchoring events exhibited an intermediate phase (Phase 2, red) in which the MT makes a transient, unstable contact with the SSN. In Phase 2, the current is partially reduced and exhibits significantly more noise and baseline drift. Phase 3 (green) depicts the characteristic current drop when the MT becomes securely anchored to the SSN. Many MT anchoring events transitioned directly from Phase 1 to Phase 3. We note that the stably anchored MT state exhibits increased low‐frequency noise compared to the open‐pore state, likely arising from thermal and electrostatic fluctuations of the MT.

### 3.2 Ionic Transport in Hybrid MT–SSNs

**FIGURE 2:**
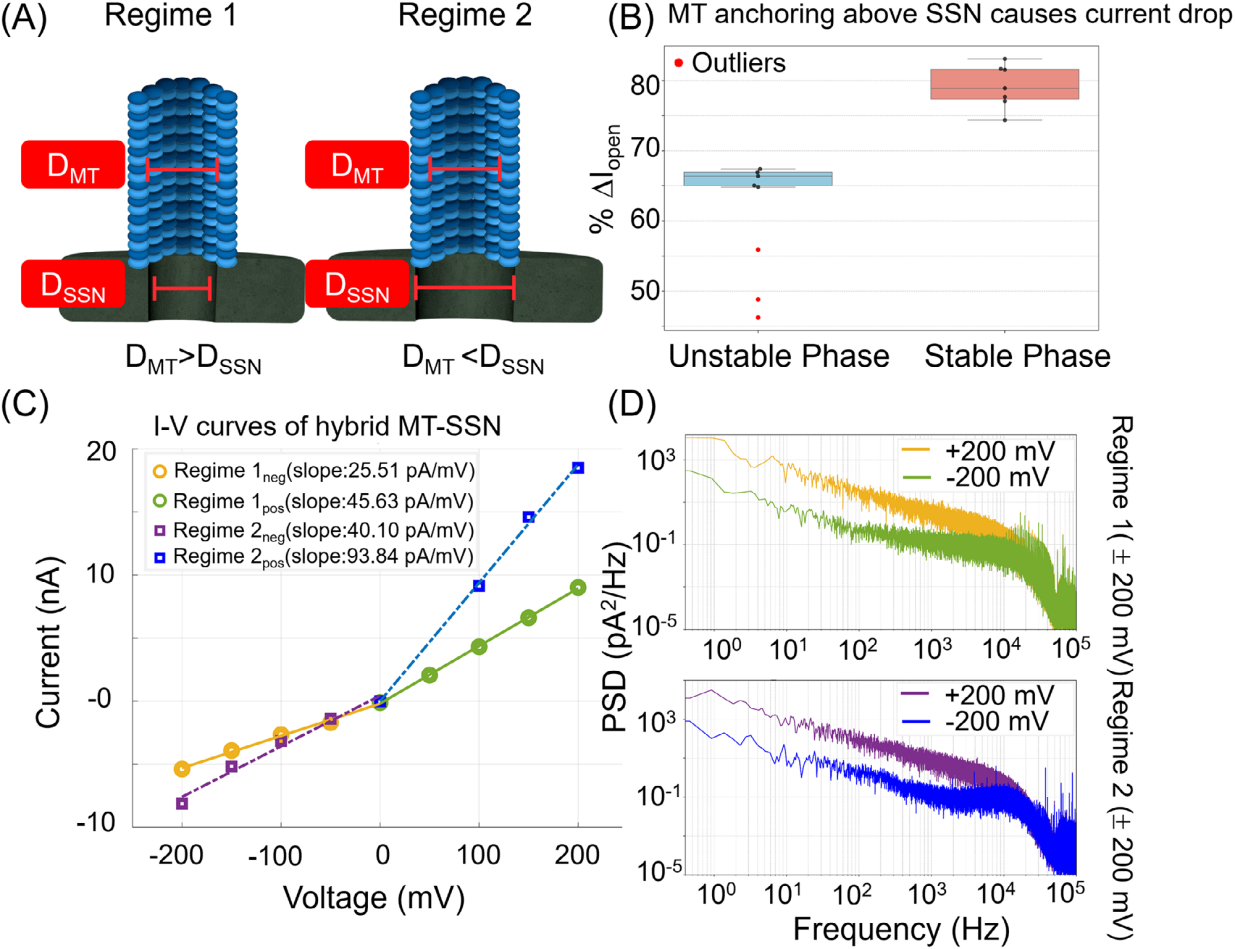
(A) Depiction of two regimes of MT–SSN configurations. In Regime 1, MT lumen diameter is larger than the SSN diameter. In Regime 2, the MT lumen diameter is smaller than the SSN diameter. (B) % Attenuation in open‐pore current upon MT anchoring to SSN in Regime 2. Two phases, intermediate/unstable anchoring (blue) and stable anchoring (red) of MTs, are shown. (C) *I*–*V* curves of hybrid MT–SSN for two regimes. Regime 2 (purple and blue) exhibits more rectification compared to Regime 1 (yellow and green). For both configurations, the *I*–*V* slopes for negative voltages are more attenuated than positive voltages, indicating an intrinsic bias in the MT–SSN for the direction of ion flow. (D) Power spectral density (PSD) plots showing noise comparison at +200 and −200 mV for Regimes 1 and 2 of hybrid MT–SSN. In both Regimes 1 and 2, the PSD level is overall higher for the negative voltage compared to the positive voltage. MT–SSN, microtubule–solid‐state nanopore.

We characterized ionic transport through the MT in two distinct regimes of hybrid MT–SSN configurations (Figure 2A). In this study, reported values represent mean ± standard deviation obtained from multiple independent MT–SSN devices and repeated translocation measurements. In Regime 1, the diameter of the SSN is smaller than the inner diameter of the MT (*D*_SSN_ < *D*_MT_), making the SSN the primary constriction site. For these experiments, we used SSNs with a diameter of 10 ± 1 nm. In Regime 2, *D*_SSN_ > *D*_MT_, where *D*_SSN_ is 16 ± 1 nm. In this second regime, the MT itself serves as the constriction site, effectively confining ionic flow to its lumen. Although Regime 1 was optimal for analyte translocation, Regime 2 provided more direct insights into ionic transport through the MT. This setup enabled the enhanced resolution of luminal transport phenomena. Both configurations demonstrated the versatility of hybrid MT–SSN systems in investigating ionic transport and single‐molecule events.

Figure 2B shows the extent to which the current is attenuated upon the anchoring of the MT to the SSN in Regime 2. For the stable anchoring of an MT onto the SSN (Figure 2B, red), a large current attenuation was observed with approximately 78.9% ± 3% attenuation in current relative to the open‐pore current. The variation in the attenuation value came from different MTs hybridized with the SSN, which have a dispersion in the length and protofilament number. During “catch‐and‐release” experiments—where an MT was inserted and rapidly ejected by voltage reversal followed by reinsertion of the MT—consistent current drops were measured for repeated anchoring of the same MT (see Section S4). On the other hand, intermediate/unstable anchoring of the MT onto the SSN (Figure 2B, blue) occurred as a transient phase during transitions into stable insertion, exhibiting significantly higher noise and baseline drift. Analyte translocation did not occur during this unstable phase, suggesting misalignment of the MT to the SSN.

Representative current–voltage (*I*–*V*) curves for the hybrid MT–SSN are shown in Figure 2C. Interestingly, in both Regimes 1 and 2, the hybrid MT–SSN exhibited a current rectification with a greater ionic current observed at positive voltages. For Regime 1, the slope of the *I*–*V* curve was 45.49 pA/mV at positive voltage and 25.51 pA/mV at negative voltage, with the positive voltage slope being 1.7 times higher. For Regime 2, the slope was 93.84 pA/mV at positive voltage and 40.10 pA/mV at negative voltage, a 2.5‐fold difference. This asymmetry in ionic transport can be attributed to a geometric asymmetry in the hybrid nanopore architecture [33], causing an asymmetry in net access resistance depending on the direction of ionic flow.

Figure 2D plots the power spectral density (PSD) of the hybrid MT–SSN at +200 and −200 mV for Regimes 1 and 2 [34]. For both of the nanopore configurations, the PSD level is overall higher for negative voltage compared to positive voltage. The increased PSD observed at negative voltages (Figure 2D) may be caused by several factors. Even though the MT remains stably anchored under negative bias, it may become less stable compared to the MT under positive bias. This could lead to greater thermal fluctuation of the MT and create charge fluctuation as the MT moves around. It has been proposed that C‐terminal tails generate oscillations in chemical potential, destabilizing ion flow and increasing current variability [35]. In contrast, positive voltages promote ion alignment with the MT's electrostatic potential, stabilizing transport and reducing noise. Future experiments aimed at probing these mechanisms directly will be essential to confirm their role in modulating current variability.

### 3.3 DNA Translocation Through Hybrid MT–SSN

**FIGURE 3:**
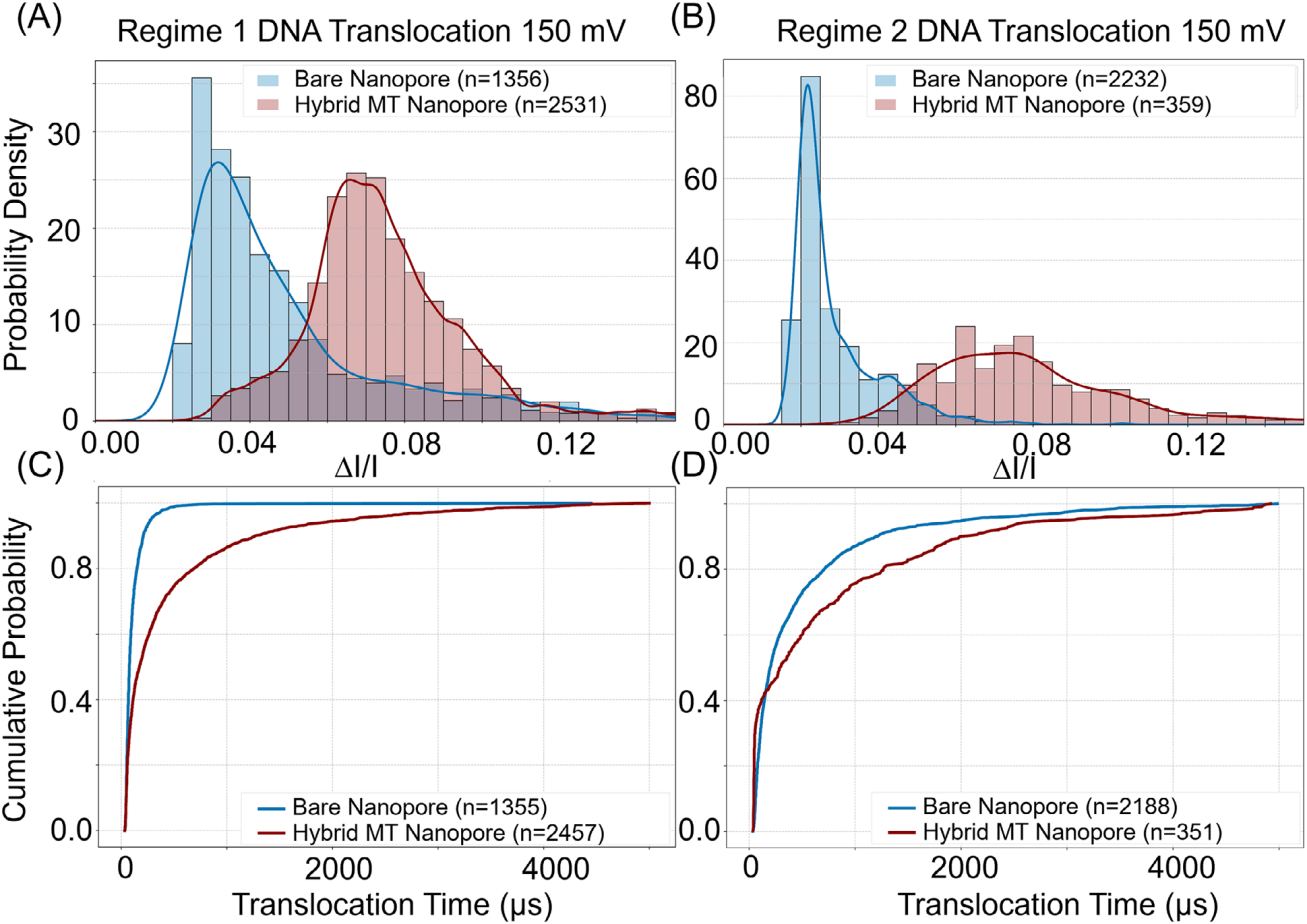
(A and B) Probability density histogram of the relative current drop (Δ*I*/*I*) during DNA translocations through a bare solid‐state nanopore (blue) and a hybrid MT–SSN (red): (A) Regimes 1 and (B) 2. The right‐shift in the histogram of the hybrid MT–SSN indicates an improved signal‐to‐noise ratio. (C and D) Cumulative probability versus DNA translocation times for a bare solid‐state nanopore (blue) and hybrid MT–SSN (red) for Regimes 1 (C) and 2 (D). The right shift of hybrid MT–SSN data indicates an increase in the number of events with slower translocation times.

We investigated the potential of the hybrid MT–SSN as a tool for label‐free, single‐molecule analysis (Figure 1F). For an analyte, 1 kb of dsDNA was used [36, 37]. The histogram of probability density of the relative current drop (Δ*I*/*I*) for DNA translocations (Figure 3A,B) was measured in Regime 1 and 2 configurations at an applied voltage of 150 mV (see Section S6 for 100 and 200 mV external voltage data). In comparison with MT‐free bare nanopores (Figure 3, blue) [3, 38], hybrid MT–SSNs (red) exhibited a rightward shift in Δ*I*/*I* for both Regimes 1 and 2, demonstrating a significantly enhanced SNR. We note that for Regime 2, where *D*_MT_ < *D*_SSN_, the number of events for DNA translocation was significantly reduced compared to Regime 1. This lower translocation rate is likely due to the steric and electrostatic repulsion of DNA from the MT base in Regime 2 configuration. In these experiments, the DNA translocated in the SSN‐to‐MT direction.

Figure 3C, the plot of cumulative probability of the translocation time (µs), shows that the temporal resolution for DNA translocation is markedly increased (right shift) upon MT hybridization onto the SSN. The characteristic translocation duration time, *τ*_tr_, which is the translocation time below which 70% of the DNA molecules translocated through MT–SSNs, is 395 µs (Regime 1) and 808 µs (Regime 2). The value of *τ*_tr_ increased by a factor of 3.5 (Regime 1) and 1.8 (Regime 2) for MT–SSN relative to bare SSN. This demonstrates that MT‐hybridized nanopores substantially improve the temporal resolution of single‐molecule translocation events.

Slower translocation time is critically important for single‐molecule analysis using nanopores because it enhances detection accuracy, which allows for inner characteristics and structures of single molecules to be resolved [39]. Furthermore, slower translocation times are critically important in nanopore experiments because they enhance detection accuracy, especially for “silent” molecules that would otherwise translocate too quickly to be resolved at the system's sampling rate [40].

**FIGURE 4:**
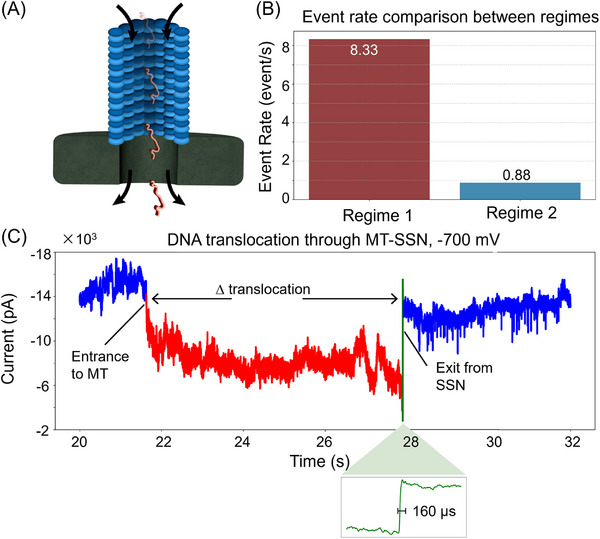
MT‐to‐SSN translocation of DNA. Schematics (A) and event rate for Regimes 1 and 2 (B). (C) Representative current trace of a DNA molecule translocating through the hybrid MT–SSN of Regime 2 at −700 mV. Blue: DNA‐free baseline current. Red: Negative current drop in the region of DNA translocation through the MT. Green: the sharp transition region as DNA translocates through and exits the SSN. The sharp transition (green) is zoomed‐in where *Δ*_translocation_ ∼ 160 µs (SSN). The total translocations occur on the scale of seconds with the translocation time *Δ*_translocation_ ∼ 6 s (MT–SSN); translocations were also observed at −400 mV (see Section S6), with *Δ*_translocation_ ∼ 20 s (MT–SSN). MT–SSN, microtubule–solid‐state nanopore.

We also conducted DNA translocation experiments in the MT‐to‐SSN direction, in which DNA first enters the MT and then passes through the SSN (Figure 4A). Figure 4B shows that the translocation event rate was markedly low compared to the SSN‐to‐MT pathway. Particularly, the event rate at Regime 2 configuration was significantly lower by 9.5‐fold than Regime 1. Threading dsDNA into a confined cylindrical channel of MT imposes a substantial entropic barrier due to the loss of configurational freedom [41, 42]. This barrier reduces the likelihood of successful capture and entry [43]. Thus, the translocation event rate substantially decreased.

Figure 4C shows a representative current trace of a single DNA molecule translocating through a Regime 2 MT–SSN at −700 mV (see Figure S8 for the −400 mV data). The current trace features two distinct phases. The initial blue segment corresponds to the hybrid pore baseline, prior to DNA capture. Upon DNA entry into the MT, a sustained current drop (red) occurs. This phase persists on the scale of seconds, reflecting the extended translocation time through the MT lumen. The green region marks the sharp current transition as DNA enters the MT/SSN interface and translocates across the SSN, completing the event. This final translocation step across the SSN has a translocation time of *Δ*_translocation_ ∼ 160 µs (zoomed‐in green region of Figure 4c). The full hybrid pore translocation takes ∼6 s at −700 mV and ∼20 s at −400 mV. For context, DNA translocation through an unmodified SSN at −200 mV occurs on the sub‐millisecond scale (mean 491 µs). The increased dwell time in the MT–SSN results from entropic and steric resistance imposed by the MT, requiring higher voltages to sustain translocation. The current traces observed were similar to previous experiments where 1 kb dsDNA translocated across asymmetric nanopores [37, 44]. We note that the distribution in length and protofilament number of MTs (w/w/o taxol) might be confounding factors to give the dispersion in translocation time.

## 4 Concluding Remarks

In this study, we demonstrate a hybrid MT–SSN platform as a proof‐of‐concept system for investigating ionic transport and single‐molecule translocation under extreme nanoscale confinement. By electrophoretically anchoring taxol‐stabilized MTs to SSNs, we form a hybrid transport pathway in which both the MT lumen and the nanopore geometry contribute to ionic conduction.

Two distinct configurations were examined, corresponding to regimes where either the SSN or the MT lumen dominates the transport constriction. These hybrid architectures exhibit pronounced current rectification and asymmetric transport behavior, reflecting the intrinsic geometric and electrostatic asymmetry of the MT–SSN system. In the configuration where the MT lumen serves as the primary constriction, ionic transport through a native biological nanotube can be directly probed.

MT anchoring introduces increased low‐frequency noise relative to the open‐pore state; however, analyte translocation events display enhanced signal contrast due to increased relative current blockade. For dsDNA, the hybrid MT–SSN substantially prolongs translocation times compared to bare SSNs, consistent with strong steric and entropic constraints imposed by the MT lumen. These long‐duration events occur at low frequencies under the present conditions and therefore represent a physical demonstration of extreme confinement rather than a high‐throughput sensing strategy. Additionally, the present configuration is most suitable for relatively short and rigid DNA fragments; capture efficiency and throughput are expected to decrease for longer or highly flexible polymers.

Overall, this work establishes a foundational framework for integrating cytoskeletal protein nanotubes with SSNs, enabling the exploration of nanoscale ionic transport and confinement‐induced slowing beyond conventional nanopore architectures. Further optimization of alignment stability and capture efficiency, and extension to single‐stranded DNA, will be required to extend this approach toward broader analytical applications.

## Conflicts of Interest

The authors declare no conflicts of interest.

## Supporting information



List here briefly the contents of the supporting information file, which will be a single PDF file containing any Supporting Information tables and figures.**Supporting File**:elps70091‐sup‐0001‐SuppMat.pdf.

## Data Availability

The data that support the findings of this study are available from the corresponding author upon reasonable request.
