# Supplementary material for: Hybrid Microtubule–Solid‐State Nanopores for Single‐Molecule Analysis
Source: Electrophoresis. 2026 Mar 29;47(5):406–13. doi: 10.1002/elps.70091 (PMC13156922; doi:10.1002/elps.70091)
Supplement: Supplementary file 1 — List here briefly the contents of the supporting information file, which will be a single PDF file containing any Supporting Information tables and figures.Supporting File:elps70091‐sup‐0001‐SuppMat.pdf. [file ELPS-47--s001.pdf]

Supplementary Information

SI-1. Current–Voltage (IV) Characterization of Solid-State Nanopores

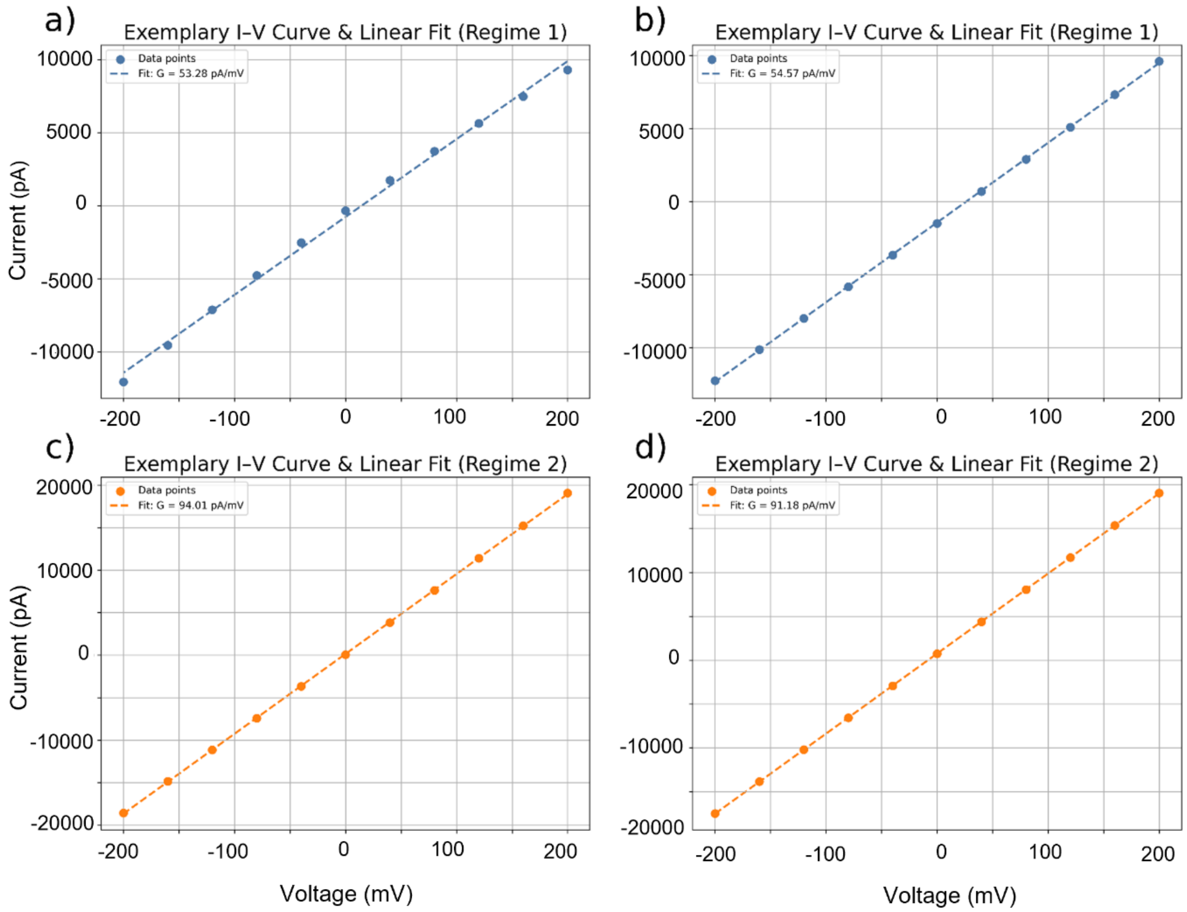

Figure S1: IV curves of the bare solid-state nanopores used in this study. Measurements were performed in both 1 M to confirm ohmic behavior prior to microtubule insertion. These curves serve as baseline controls to validate that any rectification observed after insertion is attributable to the microtubule.

SI-2: Baseline of Nanopores Prior to Microtubule Insertion

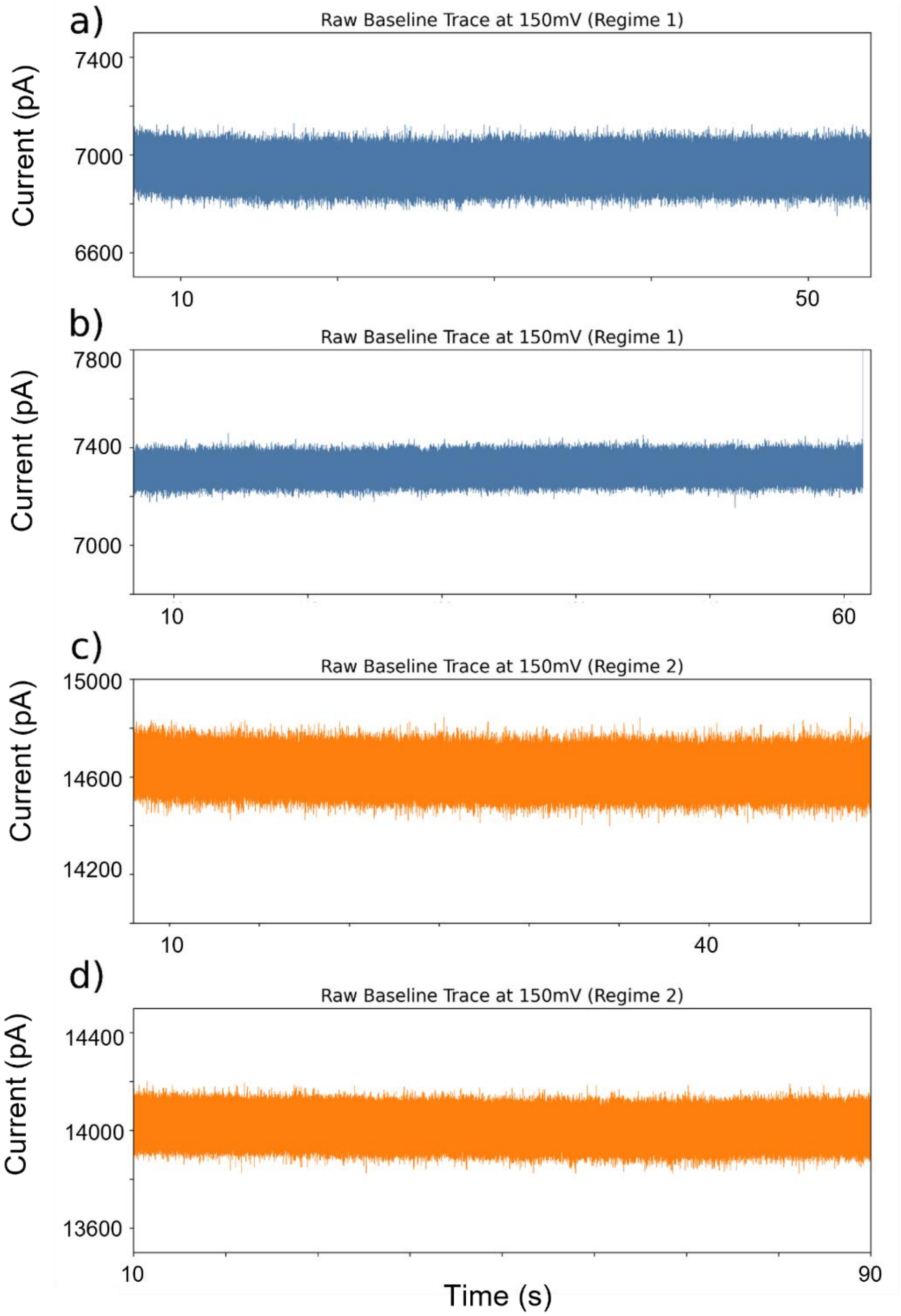

Figure S2: Representative traces of ionic current through bare nanopores at 150mV prior to microtubule insertion. These traces confirm the stability of the open pore and the absence of spontaneous clogging, false events, or baseline drift under applied voltage. (a-b) Regime 1 pores, (c-d) Regime 2 pores.

SI-3: Stable Microtubule Anchoring Events

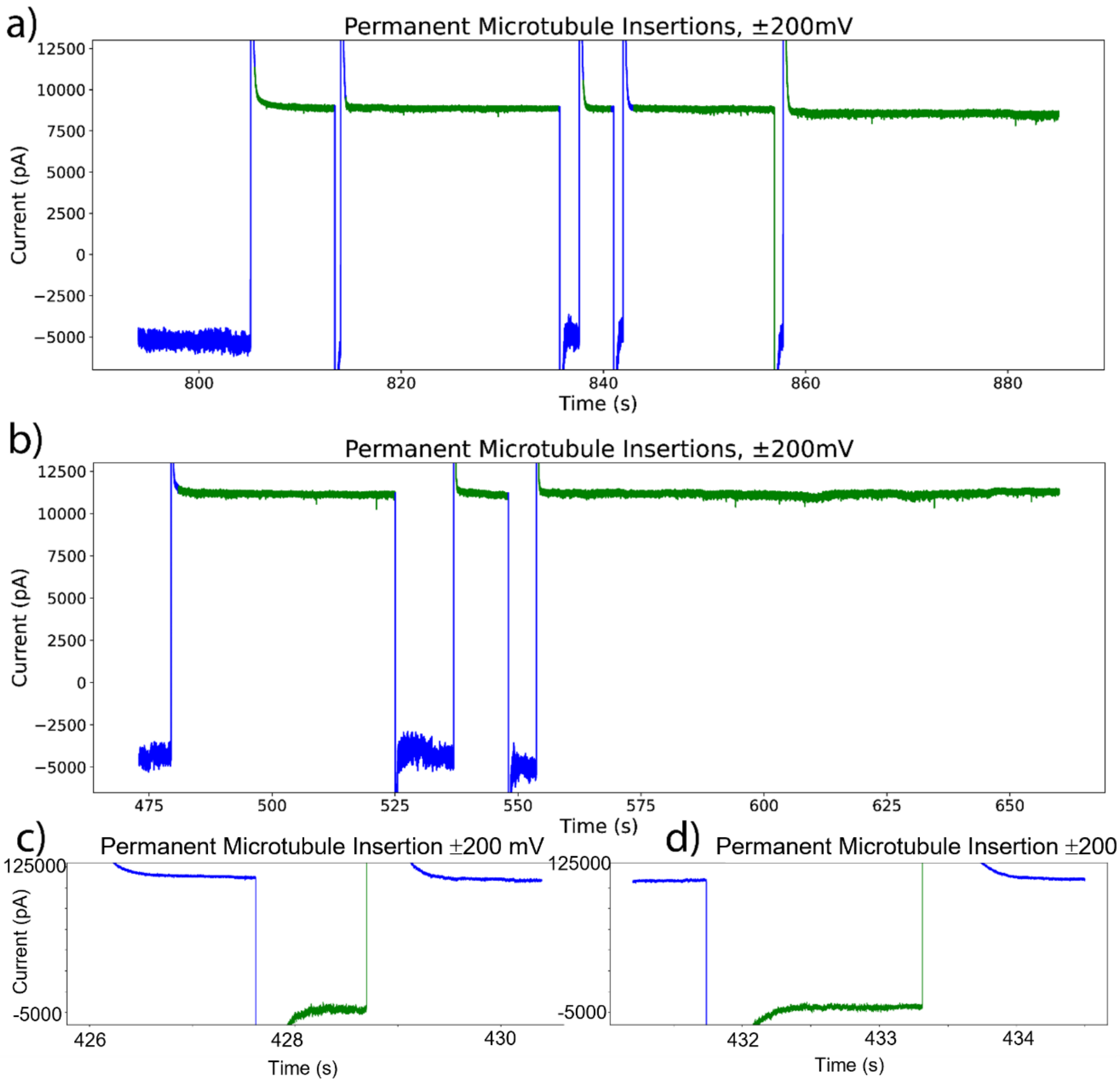

Figure S3: Consecutive voltage reversals demonstrate that the microtubule remains stably anchored in the nanopore, even after polarity reversals. This is fundamentally distinct from clogging: there is no current delay or baseline disruption. The asymmetric current behavior (increased current flow and decreased noise at positive voltages, decreased current flow and increased noise at negative voltages) can be seen. The transient spikes upon switching voltage polarity are capacitive are capacitive artifacts, likely arising from the dielectric properties of the solid-state nanopore and membrane.

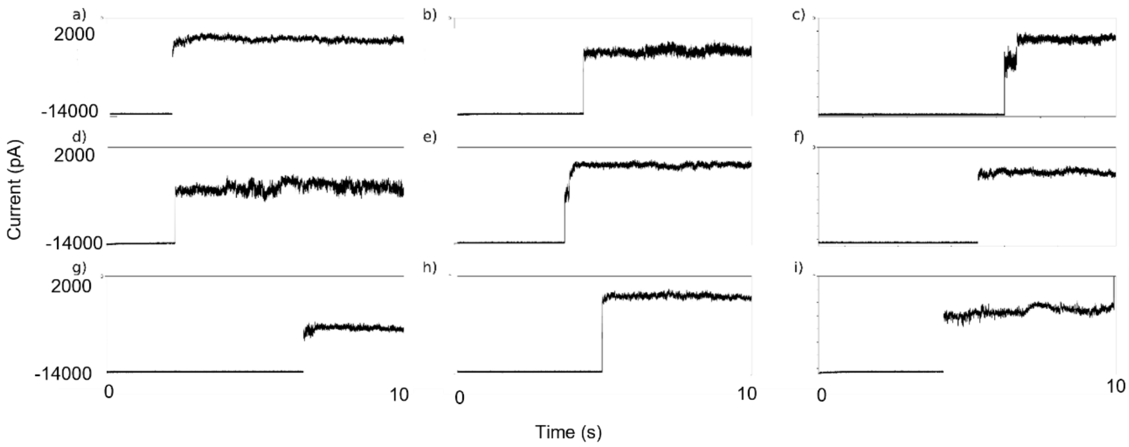

Figure S4: (a-i) Examples of permanent microtubule anchoring events in separate experiments. Stepwise decreases in current, characteristic of microtubule insertion, were remarkably consistent across trials. All recordings were performed at -150 mV.

SI-4: Reversible Microtubule Insertions

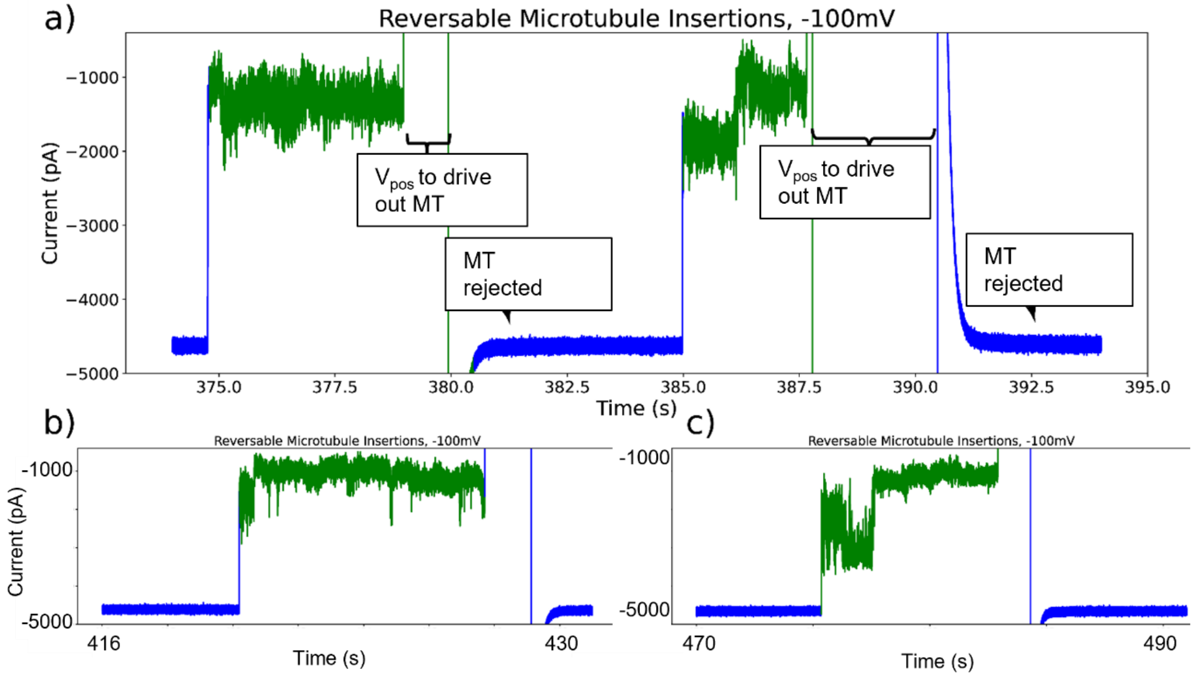

Figure S5: (a-c) Selected examples of reversible microtubule insertions. Rapid voltage reversal within ~5 seconds leads to ejection of the microtubule, distinguishing these as reversible rather than permanent events. Stepwise current restoration confirms full retraction of the microtubule from the pore.

SI-6: DNA Translocation Raw Baseline

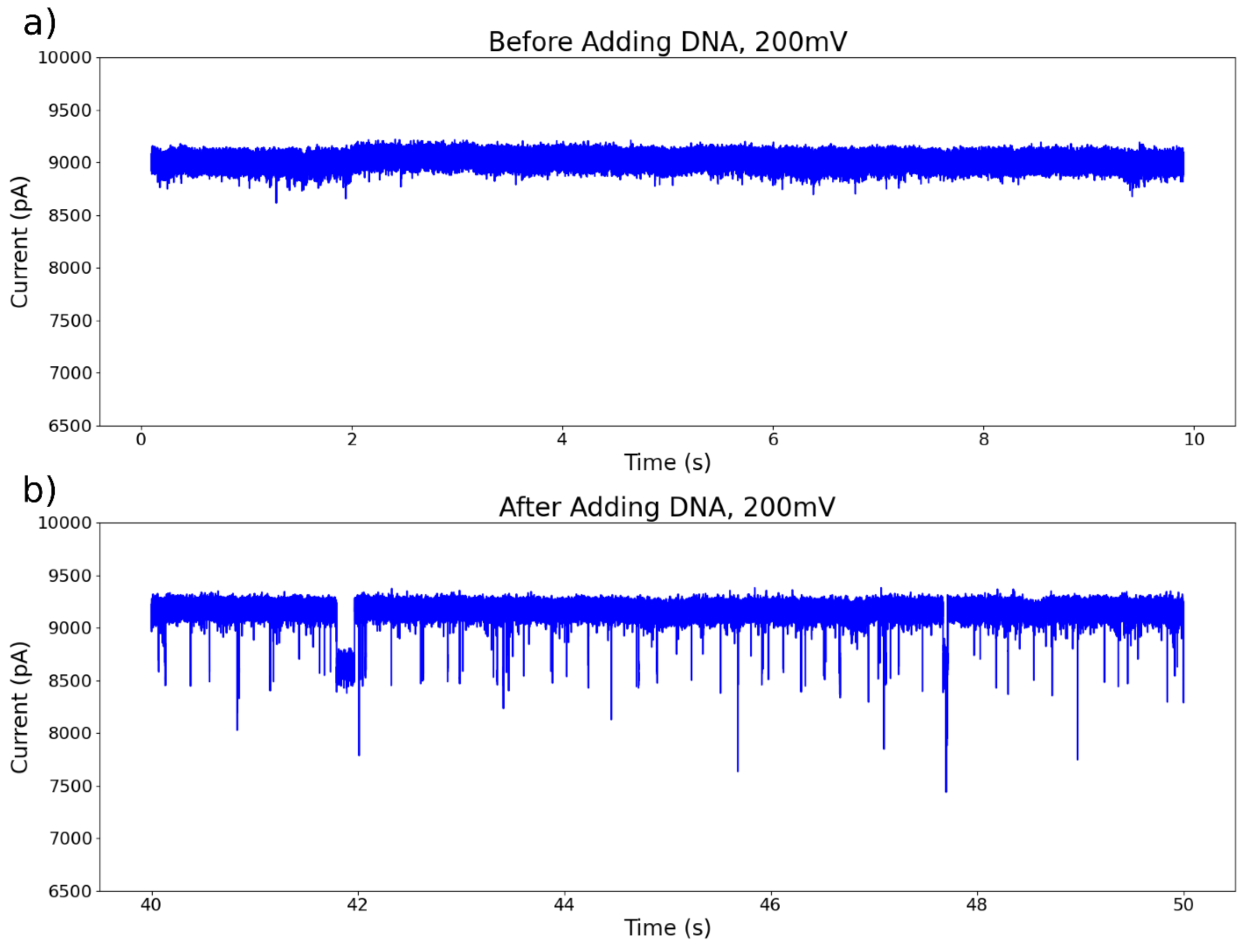

Figure S6: (a) Current trace of the hybrid MT-SSN at +200 mV before introduction of DNA, showing a clean hybrid microtubule–nanopore baseline. (b) Current trace after DNA addition at +200 mV. Resistive spikes are consistent with translocation of double-stranded DNA through the hybrid nanopore.

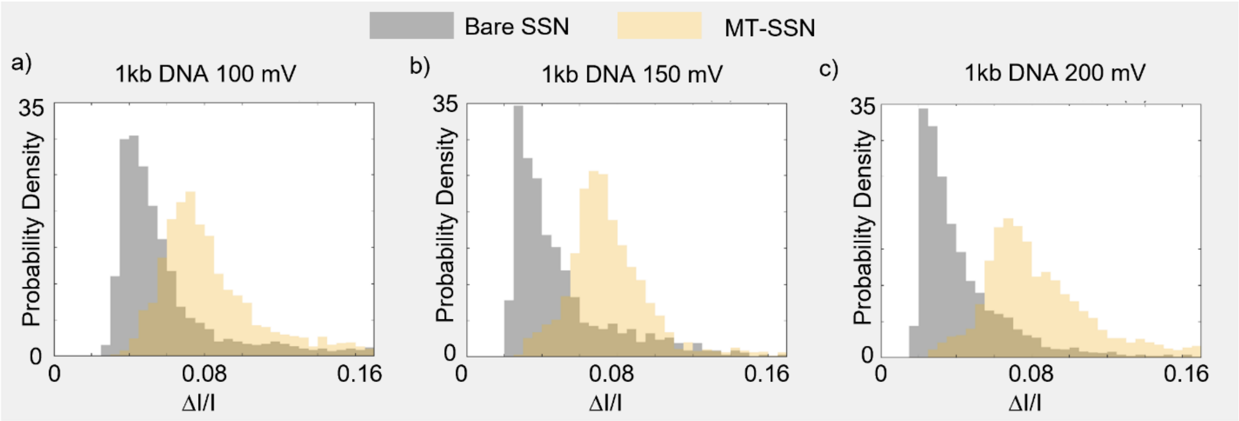

Figure S7: Alternate Experiment at (a) 100mV, (b) 150mV, and (c) 200mV showing a similarly increased SNR with the hybrid microtubule nanopore when translocating dsDNA

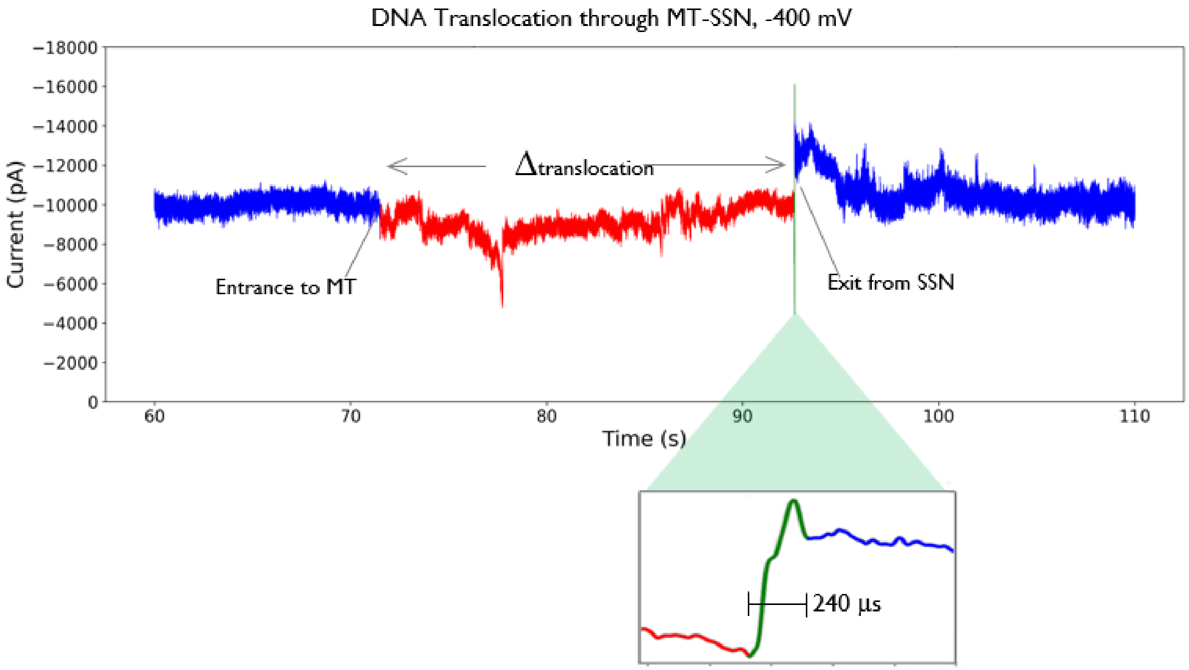

Figure S8: MT-to-SSN translocation of DNA through the hybrid MT-SSN at -400mV. Blue: DNA-free baseline current. Red: the region of DNA translocation through the MT. Green: the sharp transition region of DNA translocation through the SSN. The sharp transition (green) is zoomed-in where $\Delta_{translocation}$ ~ 240 µs. The translocations occur on the scale of seconds with the translocation time ($\Delta_{translocation}$) ~20 sec.

SI-7: Relative Changes In Voltage-Current Responses of Hybrid MT-SSN Nanopores

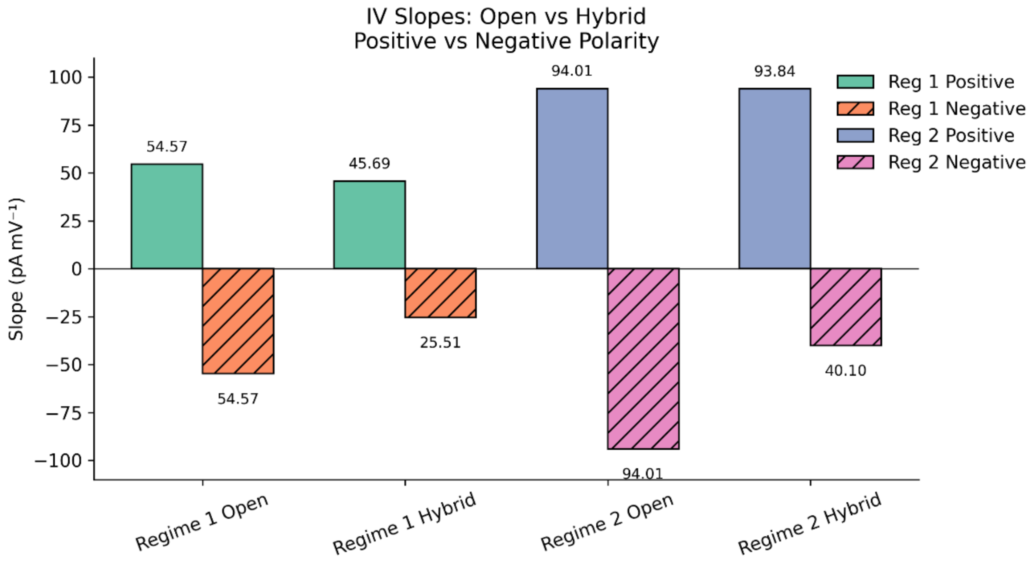

Figure S9: I-V slope magnitudes of the hybrid MT-SSNs for both Regime 1 and Regime 2. In both regimes, asymmetry is present between positive and negative applied voltages.

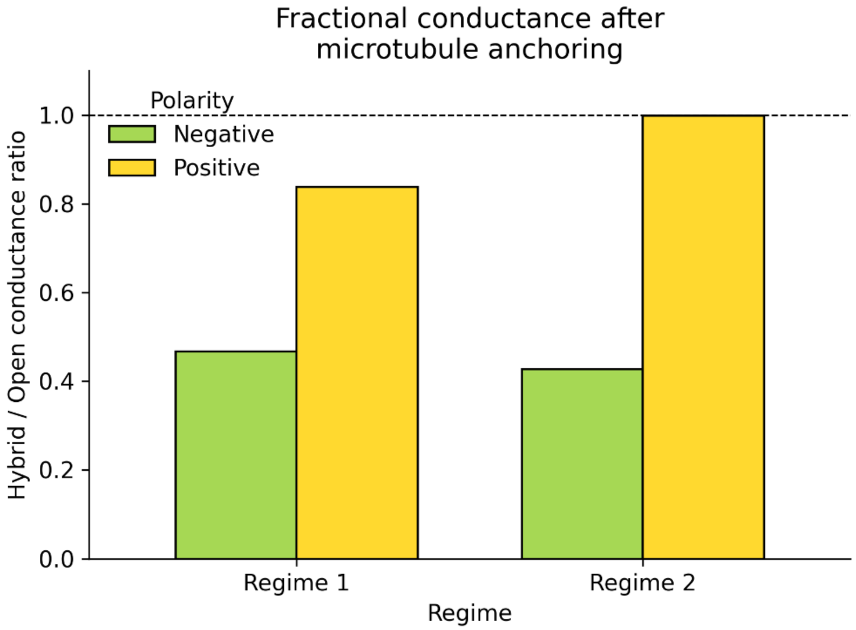

Figure S10: *Hybrid-to-open conductance rations of the pores under positive and negative bias across Regime 1 and Regime 2 configurations. Both hybrid MT-SSNs experience a similar reduction in current at negative voltages.*
